# Suppression and resurgence: the evolving epidemiology of seasonal influenza from 2015 to 2024 in a core urban district of Beijing, China

**DOI:** 10.3389/fpubh.2026.1800701

**Published:** 2026-05-14

**Authors:** Xing Gao, Panpan Qin, Xiao Qi, Yanli Wan, Zhiyuan Xu, Hongpu Hu

**Affiliations:** 1 Institute of Medical Information/Medical Library, Chinese Academy of Medical Sciences & Peking Union Medical College, Beijing, China; 2 Center for Disease Control and Prevention of Chaoyang District, Beijing, China

**Keywords:** immunity gap, influenza, interrupted time series analysis, resurgence, sentinel surveillance

## Abstract

**Background:**

The COVID-19 pandemic significantly disrupted seasonal influenza dynamics. Understanding post-pandemic rebound patterns is crucial for optimizing future public health strategies.

**Methods:**

We conducted a comprehensive epidemiological analysis of multi-source surveillance data collected from Chaoyang District, Beijing, China, a representative core urban area, spanning 2015 to 2024. Data sources included: Influenza-like illness (ILI) surveillance records, virological results, and reported influenza cases. A segmented interrupted time-series (ITS) framework, utilizing generalized additive mixed models (GAMMs) with negative binomial distribution and AR (1) structure, characterized the disruption and post-pandemic shifts. This robust modeling approach quantified deviations from historical baselines. We also characterized epidemic seasonality, viral strain dominance, and calculated age-stratified rate ratios (RRs).

**Results:**

Analysis of 2,468,817 ILI cases revealed a distinct “increase-suppression-resurgence” pattern. The annual proportion of influenza-like illness (ILI%) exhibited a peak of 3.85% in 2019, subsequently declined to 2.29% during 2020–2022, and then rebounded to 4.42% in 2024. Segmented GAMM-AR(1) modeling demonstrated that pandemic-era ILI% remained consistently below counterfactual projections, succeeded by a substantial post-pandemic rebound (RR = 1.72, *p* < 0.001). The models also indicated a structural shift from stable historical seasonality toward highly non-linear temporal dynamics (edf = 9.19, *p* < 001), where typical winter epidemics were replaced by irregular outbreaks, followed by a prolonged 28-week season in 2023–2024. Virological surveillance consistently showed the absence of B/Yamagata lineage detection after 2020. Notably, while children maintained the highest absolute incidence, adults (15–64 years) exhibited the most profound post-pandemic surge in relative risk (RR = 8.43 vs. 2016–2019 baseline), significantly outpacing people over 65 (RR = 3.53) and children (RR = 3.28).

**Conclusion:**

The COVID-19 pandemic was associated with a complex shift in influenza epidemiology, characterized by intensified post-pandemic activity, altered seasonality, and a disproportionate redistribution of reported burden toward working-age adults. This demographic shift likely reflects a combination of post-pandemic changes in healthcare-seeking behavior, surveillance sensitivity, and host-level biological factors, with potential contributions from population-level immune waning. The sustained absence of B/Yamagata lineage detections aligns with global evidence of its probable extinction. These findings underscore the importance of age-stratified influenza monitoring and highlight the need for integrated serological, virological, and behavioral studies to elucidate the determinants of post-pandemic influenza burden.

## 1 Introduction

Seasonal influenza has traditionally exhibited stable winter–spring seasonality, shaped by climatic conditions and population immunity (1–4). The emergence of SARS-CoV-2 and the widespread implementation of non-pharmaceutical interventions (NPIs) disrupted this pattern, leading to an unprecedented and prolonged suppression of influenza circulation (3, 5–8). With the relaxation of NPIs and the transition away from the acute phase of the COVID-19 pandemic, influenza activity has re-emerged (6, 9–11). However, this resurgence has not simply mirrored pre-pandemic patterns (12). Unprecedented shifts, particularly the apparent global cessation of the B/Yamagata lineage, have further increased uncertainty in the post-pandemic epidemiological landscape. This development prompts a critical assessment of whether influenza dynamics are reverting to historical norms or transitioning toward a distinct transmission regime (5, 13, 14).

Most existing studies have examined influenza trends at global or national scales, offering valuable insights into broad temporal patterns (9, 10, 15). However, these aggregated analyses often obscure substantial heterogeneity within urban populations, where high population density and complex mobility structures drive distinct transmission dynamics. Consequently, district-level analyses are essential to capture localized epidemiological patterns and facilitate precise public health surveillance in highly urbanized settings.

As the largest central district of Beijing, Chaoyang covers an area of 470.8 km^2^ and has a population of approximately 3.45 million, representing a highly urbanized and densely populated metropolitan setting (16). The district is characterized by a heterogeneous spatial structure in which central business districts, residential neighborhoods, and peri-urban areas coexist alongside major transportation hubs. This combination of high population density and intense human mobility provides a suitable context for examining the transmission and diffusion dynamics of influenza in an urban environment. Leveraging continuous surveillance data from 2015 to 2024 in this critical region, the present study characterizes post-pandemic influenza dynamics across three specific dimensions.

(1) Has the intensity of influenza epidemics returned to or exceeded historical baselines?
(2) Have the temporal characteristics, such as onset timing, duration, and viral strain alternation, been fundamentally altered?
(3) Has the burden of infection shifted across age groups, suggesting a restructuring of population susceptibility?

## 2 Materials and methods

### 2.1 Study area

Chaoyang District (39°49′–40°05′N, 116°21′–116°38′E) is characterized by a temperate semi-humid continental monsoon climate. This climatic setting exhibits distinct seasonality with hot, rainy summers and cold, dry winters, providing the environmental context for seasonal influenza transmission (16).

### 2.2 Data collection

Surveillance data were integrated from three complementary monitoring systems managed by the Chaoyang District Center for Disease Control and Prevention.

#### 2.2.1 Syndromic surveillance

Aggregated influenza-like illness (ILI) data, which serve as a syndromic proxy for acute respiratory infections, were retrieved from the Beijing Hospital Infectious Disease Surveillance and Warning Information System, covering a network of 26 medical institutions (12 tertiary, 11 secondary, and 3 primary hospitals). An ILI case was defined as a patient with fever (axillary temperature ≥ 38 °C) and either cough or sore throat (17, 18). We extracted weekly counts of ILI cases alongside total outpatient and emergency visits. This dataset covers the period from week 1 of 2015 to week 52 of 2024.

#### 2.2.2 Virological surveillance

Weekly virological data were sourced from the Chinese National Influenza Surveillance Information System (CNISIS). The district surveillance network comprised three national-level sentinel hospitals (Beijing Chaoyang Hospital, China-Japan Friendship Hospital, and the Capital Institute of Pediatrics). Following the National Influenza Surveillance Technical Guidelines (2017 Edition) (17), each sentinel site collected 20 throat swab specimens per week from ILI patients with illness onset within 3 days. Specimens were transported at 4 °C and delivered to network laboratories within 24 h of collection, where they were tested using real-time RT-PCR and viral culture for influenza A and B viruses (19). Due to system implementation timelines, virological data were available from week 1 of 2016 to week 52 of 2024.

#### 2.2.3 Case reporting and demographics

Notifiable influenza cases, comprising both clinically diagnosed and laboratory-confirmed infections, were extracted from the China Information System for Disease Control and Prevention (CISDCP) (20, 21). The dataset was restricted to cases with residential addresses in Chaoyang District and onset dates ranging from week 1 of 2016 to week 52 of 2024. According to the national Diagnostic Criteria for Influenza (WS 285-2008) (22), a clinically diagnosed case was defined as a patient with epidemiological exposure during an influenza outbreak and at least one compatible clinical manifestation, including acute-onset fever with systemic symptoms (chills, headache, myalgia, or fatigue) and/or respiratory symptoms (cough or sore throat); a laboratory-confirmed case was defined as an influenza-like illness or clinically diagnosed case with at least one positive laboratory result, including viral isolation, influenza-specific nucleic acid detection by RT-PCR, antigen detection, or a ≥4-fold rise in influenza-specific antibody titers between paired acute and convalescent sera (22, 23). To serve as denominators for incidence rate calculations, annual population data were retrieved from the Beijing Chaoyang District Statistical Yearbook (24).

### 2.3 Statistical analysis

#### 2.3.1 Interrupted time series design

We used an interrupted time series (ITS) design to evaluate temporal changes in ILI activity in relation to major COVID-19 policy interventions. The primary outcome was the weekly number of outpatient and emergency ILI visits, with the corresponding total number of outpatient and emergency visits used to construct the proportion of ILI. The study period was divided into three epidemiological phases based on the timing of key non-pharmaceutical intervention (NPI) policies (25, 26): (1) pre-pandemic (before week 4 of 2020; pre-Wuhan lockdown), (2) pandemic (week 4 of 2020 to week 48 of 2022; strict NPI implementation), and (3) post-pandemic (week 49 of 2022 onwards; following the “New Ten Measures”).

#### 2.3.2 Segmented generalized additive mixed model specification

To model the temporal dynamics of ILI, we specified a segmented generalized additive mixed model (GAMM) implemented in R (mgcv package). Weekly ILI counts were assumed to follow a negative binomial distribution to accommodate overdispersion, which was supported by standard diagnostic checks. The expected weekly count of ILI cases, $E[Y_{t}]$ , was modeled as:


$$\log(E[Y_{t}])=\beta_{0}+\beta_{\text{phase}}+f{\left(t\right)}_{\text{phase}}+s(\text{week})+\log(N_{t})$$

where $\beta_{0}$ is the global intercept and $\beta_{\text{phase}}$ denotes phase-specific intercepts capturing level changes between the three epidemiological phases. To characterize non-linear long-term trends independent of seasonality, we included $f{\left(t\right)}_{\text{phase}}$ , a thin plate regression spline for calendar time stratified by phase. The basis dimension ( k ) for the long-term trend splines was set to 25, and its adequacy was subsequently verified using the standard gam.check() procedure. Intrinsic annual seasonality was modeled using s(week) , a cyclic cubic regression spline with 52 knots to ensure continuity across calendar years. The natural logarithm of the total number of outpatient and emergency visits, $\log(N_{t})$ , was included as an offset term to estimate the dynamics of the proportion of ILI.

To account for temporal autocorrelation inherent in infectious disease time series, we incorporated a first-order autoregressive [AR (1)] correlation structure into the GAMM.

#### 2.3.3 Model diagnostics and sensitivity analyses

We assessed overall model fit by inspecting standardized residuals, quantile–quantile plots, and the distribution of residuals under the assumed negative binomial error structure.

To further evaluate residual autocorrelation, we examined autocorrelation (ACF) and partial autocorrelation (PACF) functions alongside Durbin–Watson tests, confirming that the inclusion of the AR (1) structure effectively mitigated serial correlation.

Smooth terms for the phase-specific long-term trend and intrinsic seasonality were fitted using penalized regression splines, with smoothing parameters selected via restricted maximum likelihood (REML). The effective degrees of freedom and basis adequacy tests were examined to ensure that the fitted smooth functions achieved an optimal balance between flexibility and smoothness.

To test the robustness of the predefined phase boundaries (pandemic start: week 4 of 2020; post-pandemic start: week 49 of 2022), we conducted sensitivity analyses by shifting each boundary forward and backward by up to 2 weeks. This approach allowed us to assess whether the estimated changes in the proportion of ILI were sensitive to small perturbations in the assumed timing of policy transitions.

#### 2.3.4 Counterfactual scenarios and immunity gap estimation

To quantify the potential “immunity gap” accumulated during the period of strict NPI implementation, we constructed a counterfactual scenario for the years 2020–2024. This simulation aimed to reconstruct the hypothetical “historical norm” trajectory of the proportion of ILI in the absence of pandemic interventions. The projection was generated by retaining the intrinsic seasonal spline, s(week), and the global intercept derived from the pre-pandemic phase, while constraining phase-specific coefficients and pandemic-related trend terms to zero. The difference between this counterfactual baseline and the observed the proportion of ILI was used as a proxy for the population-level susceptibility deficit. It is important to note that while this ‘immunity gap’ provides a schematic visualization of reduced population exposure during the pandemic period, it does not correspond to a formally estimated model parameter. Consistent with its lack of serological validation, it should be interpreted as an epidemiologically inferred construct rather than a biologically quantified metric.

#### 2.3.5 Characterization of seasonality and circulating strains

Seasonality metrics were calculated over a surveillance year spanning from week 27 to the following week 26 (27). Epidemic seasons were delineated using a fixed 10% positivity threshold, which is commonly utilized in virological surveillance to capture sustained community transmission (The onset of an epidemic season was defined as the first of at least two consecutive weeks with a positive rate ≥10%). Conversely, the end was marked by the week preceding two consecutive weeks where the rate fell below 10%. The duration was calculated as the interval between the onset and end weeks. The peak was identified as the week exhibiting the highest positivity rate within the epidemic period. To assess the robustness of our epidemic season definitions, we conducted a sensitivity analysis using alternative thresholds of 5 and 15%.

To characterize the circulating strains, the evolution of viral composition was analyzed by calculating weekly constituent ratios to visualize shifts in strain dominance.

#### 2.3.6 Age-stratified disease burden

To assess demographic shifts in influenza burden, we stratified the population into four age groups: 0–4, 5–14, 15–64, and ≥65 years. The temporal evolution of age distribution was evaluated by monitoring the annual proportional contribution of each cohort. We quantified changes in risk by comparing phase-specific incidence rates (per 100,000 population). Rate Ratios (RRs) were calculated relative to the pre-pandemic baseline, with 95% confidence intervals (CIs) estimated assuming a Poisson distribution. Deviations in disease burden were visualized using lollipop charts. To ensure symmetrical representation of reciprocal changes (i.e., suppression versus resurgence), the RRs were plotted on a $\log_{10}$ scale.

#### 2.3.7 Data management and statistical software

The Generalized Additive Models were implemented using the mgcv package within the R statistical environment (version 4.5.2). Prior to modeling, data cleaning, descriptive statistics, and the generation of figures were conducted using Python (version 3.7), relying on the pandas, NumPy, and matplotlib libraries.

## 3 Results

### 3.1 Disruption of seasonal influenza intensity and temporal dynamics

From 2015 to 2024, a total of 74,861,814 outpatient and emergency visits were recorded. Of these, 2,468,817 were identified as ILI cases, corresponding to an overall proportion of ILI of 3.30% (Table 1).

**Table 1:** Surveillance of influenza-like illness in sentinel hospitals in the Chaoyang District in Beijing, China, 2015–2024.

Year	Number of influenza-like cases	Number of outpatient and emergency cases	Proportion of influenza-like cases (%)	Dominant circulating strain(s)
2015	155,901	7,453,320	2.11	–
2016	193,989	8,072,105	2.42	A(H3N2)
2017	225,315	7,448,204	2.97	A(H3N2)
2018	271,143	7,836,805	3.43	A(H1N1)pdm09
2019	313,771	8,045,808	3.85	B/Victoria
2020	119,452	4,780,245	2.29	A(H3N2)
2021	177,003	6,727,614	2.56	B/Victoria
2022	171,178	6,166,023	2.65	B/Victoria
2023	422,423	9,024,969	4.22	A(H3N2)
2024	418,642	9,306,721	4.42	B/Victoria and A(H1N1)pdm09
Total	2,468,817	74,861,814	3.30	–

“–” indicates that data were not available or could not be determined. Detailed virological subtyping data in the current dataset were only accessible from Week 51 of 2015 onwards. Consequently, the dominant strain for the entire year 2015, and a combined dominant strain for the “Total” row across all years, could not be conclusively identified.

Pre-pandemic activity exhibited a stable seasonal pattern with gradually increasing intensity, rising from 2.11% in 2015 to a peak of 3.85% in 2019. With the implementation of COVID-19 interventions in early 2020, the proportion of ILI dropped precipitously to 2.29% and remained suppressed at historical lows (2.29–2.65%) throughout the pandemic phase. Post-pandemic trends reversed this decline, as the proportion of ILI rebounded to 4.22% in 2023 and climbed to a study-period high of 4.42% in 2024, surpassing all pre-pandemic records (Table 1).

**Figure 1:**
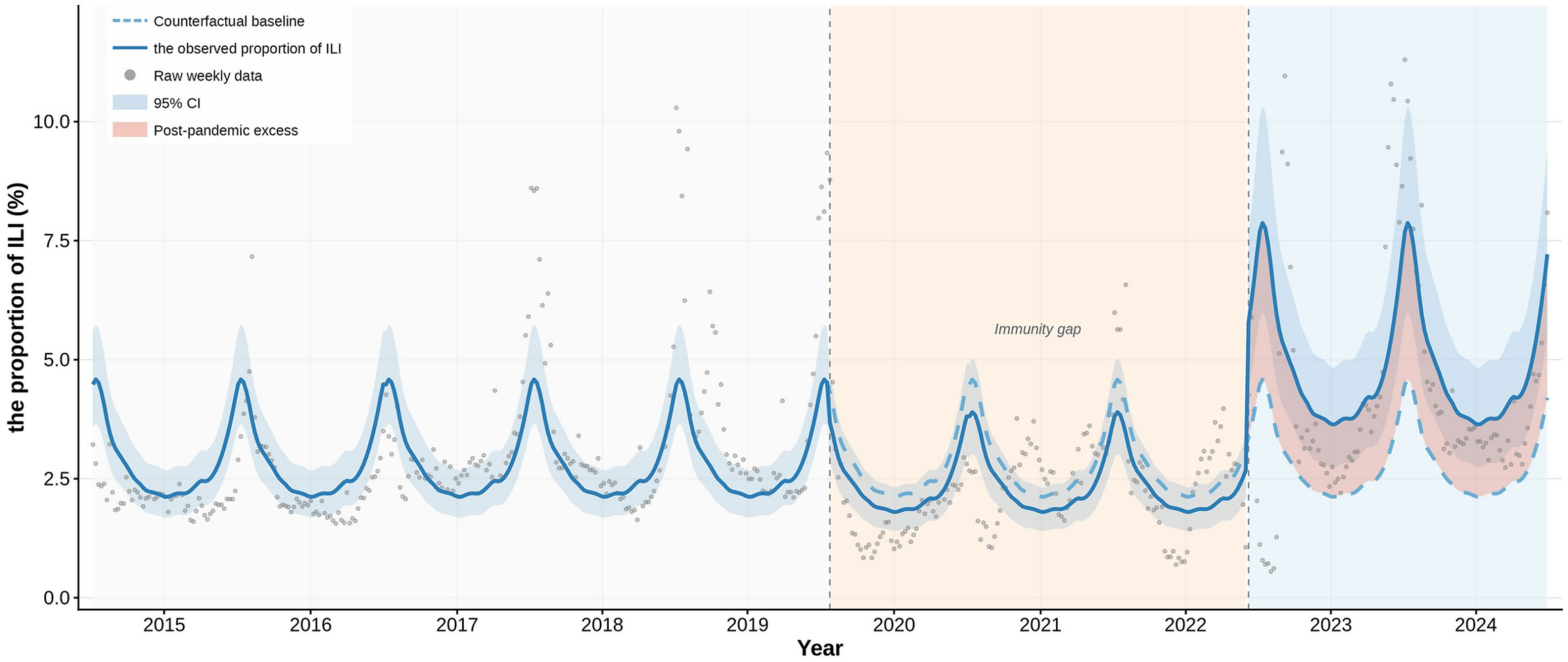
Segmented GAMM-AR (1) modeling of the observed proportion of influenza-like illness (ILI) versus the model-derived counterfactual baseline (2015–2024). This figure presents the observed weekly proportion of influenza-like illness (ILI) in comparison to a model-derived counterfactual baseline, illustrating the epidemiological impact of the COVID-19 pandemic across different phases from 2015 to 2024. The solid dark blue line represents the model-fitted trend of the observed weekly ILI proportion, with grey dots indicating the raw weekly data. The dashed light blue line denotes the counterfactual baseline, established using a generalized additive mixed model with an autoregressive AR (1) structure, which simulates the expected ILI trajectory assuming the absence of the COVID-19 pandemic. The light blue band represents the 95% confidence interval (CI) of this baseline. Vertical dashed lines demarcate the study period into three distinct epidemiological phases: pre-pandemic (unshaded), pandemic (shaded in light orange), and post-pandemic (shaded in light blue). During the pandemic phase, the substantial suppression of the observed ILI proportion below the expected baseline created an estimated immunity gap (highlighted by the grey word). Subsequently, in the post-pandemic phase, the red shaded area illustrates the post-pandemic excess, quantifying the magnitude by which the observed proportion of ILI resurgence surpassed the historical counterfactual baseline. Note: The annotation of the “immunity gap” serves as a schematic visualization of reduced population exposure and accumulated susceptibility during the pandemic. It is a model-inferred epidemiological concept representing accumulated susceptibility rather than a quantitatively estimated model parameter, and it was not directly measured via serological data.

The interrupted time series analysis using the GAMM–AR (1) model confirmed a distinct deviation between the observed proportion of ILI and the counterfactual trajectory expected in the absence of the pandemic (Figure 1). During the pandemic phase (2020–2022), the proportion of ILI did not significantly differ from the pre-pandemic counterfactual baseline (RR = 0.852, 95% CI: 0.683–1.063, *p* = 0.157; Table 2), reflecting the suppressive effect of non-pharmaceutical interventions on respiratory pathogen transmission. In contrast, the post-pandemic phase (2023–2024) was characterized by a substantial and statistically significant rebound, with the proportion of ILI 72.3% higher than the pre-pandemic counterfactual level (RR = 1.723, 95% CI: 1.324–2.242, *p* < 0.001; Table 2). The underlying seasonal periodicity of ILI was highly significant throughout the study period (edf = 9.19, *p* < 0.001; Table 2).

**Table 2:** Phase-specific rate ratios (RR) of influenza-like illness proportion and seasonal effect estimated by the segmented GAMM–AR(1) model.

Model term	*β* (SE)	RR (95% CI)	*p*-value
Parametric phase effects			
Pandemic vs. Pre-pandemic	−0.160 (0.113)	0.852 (0.683–1.063)	0.157
Post-pandemic vs. Pre-pandemic	0.544 (0.134)	1.723 (1.324–2.242)	<0.001
Non-parametric smooth terms	edf		
Seasonal smooth, s(week)	9.19	—	<0.001

Total N=522 weekly observations (Pre-pandemic phase: n=264 ; Pandemic phase: n=150 ; Post-pandemic phase: n=108 ). The model is a segmented negative binomial GAMM with an AR(1) correlation structure ( ϕ=0.844 ) and REML estimation. The natural logarithm of total outpatient and emergency visits was included as an offset. RR = rate ratio; CI = confidence interval; SE = standard error; edf = estimated degrees of freedom.

Comprehensive model diagnostics confirmed the adequacy of the specified segmented GAMM-AR (1) approach. The gam.check() procedure verified that the chosen basis dimensions ( k=25 ) for the phase-stratified smoothing splines were sufficient to capture non-linear trends without overfitting (all basis adequacy tests: P>0.05 ). The assumption of the negative binomial distribution was also validated, adequately capturing the overdispersion of weekly ILI counts, as confirmed by standard diagnostic checks (Supplementary Figure S1). Furthermore, incorporating the first-order autoregressive structure ( ϕ=0.844 ) successfully eliminated temporal dependencies in the residuals. The Durbin–Watson statistic improved significantly from 0.52 in the basic model to 2.18 in the GAMM, yielding residuals that closely approximated white noise, as visually corroborated by the autocorrelation (ACF) and partial autocorrelation (PACF) plots (Supplementary Figure S2). Finally, sensitivity analyses evaluating alternative phase boundary definitions (shifting the pandemic start and end dates by ±2 weeks) confirmed the robustness of the post-pandemic rebound. The estimated relative risks remained statistically significant and consistent with the primary model in four of the five alternative scenarios (Supplementary Figure S3).

### 3.2 Shifts in influenza virus distribution and seasonality

#### 3.2.1 Temporal distribution of influenza subtypes and lineages

**Figure 2:**
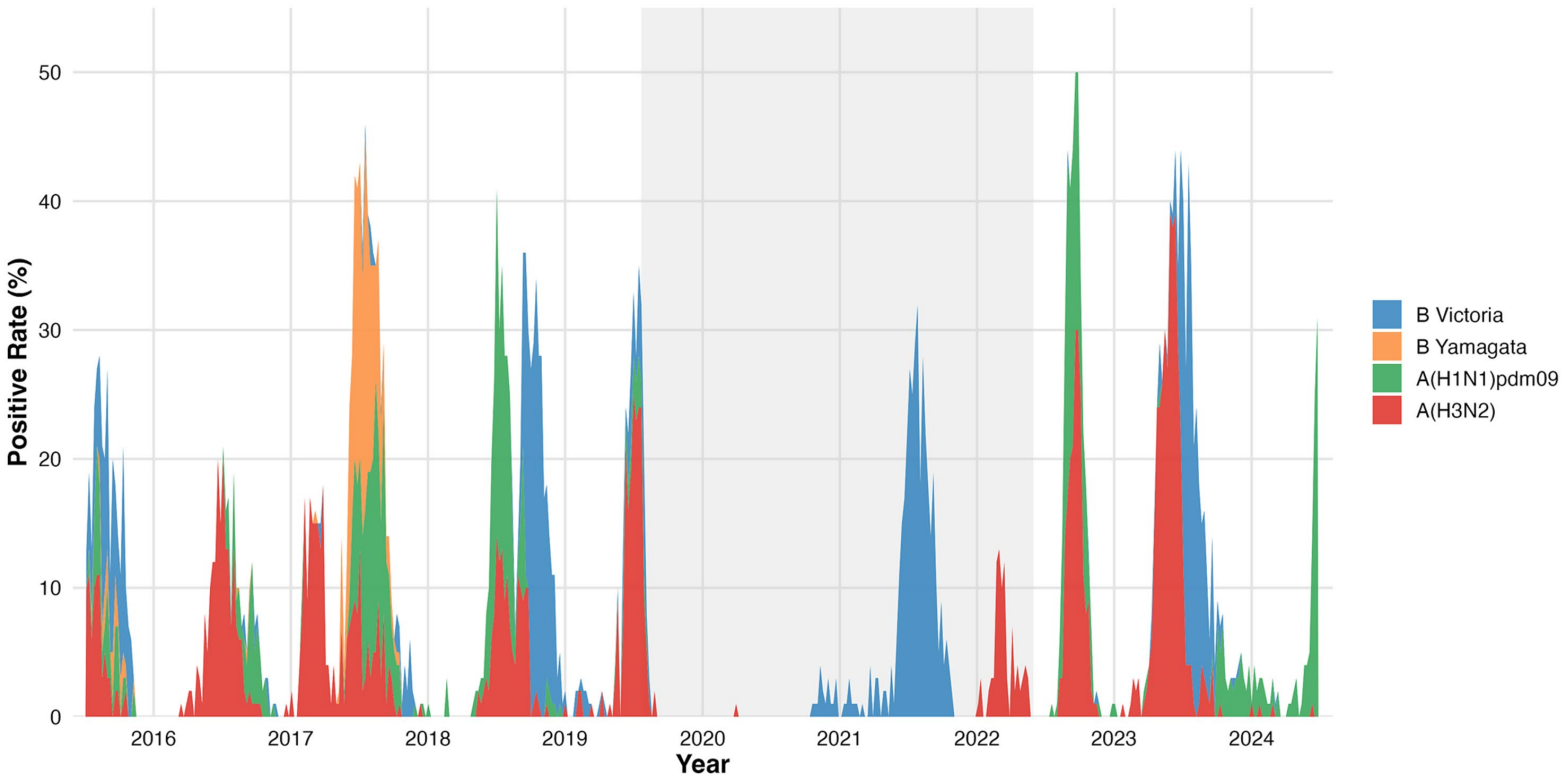
Longitudinal surveillance of circulating influenza strains and positive rates from 2016 to 2024. This figure illustrates the temporal dynamics and changing composition of circulating influenza strains, alongside their weekly positive rates, from 2016 to 2024. The *x*-axis represents the year, while the *y*-axis indicates the positive rate (%). The stacked area graph displays the weekly proportion of influenza-positive specimens attributable to each influenza subtype and lineage: dark blue for B Victoria, orange for B Yamagata, green for A(H1N1)pdm09, and red for A(H3N2). The grey shaded area highlights the COVID-19 pandemic period (approximately early 2020 to mid-2022), during which a substantial suppression in influenza activity and shifts in strain predominance were observed.

Changes in viral composition were observed over the study period (Figure 2; Supplementary Table S1). During the pre-pandemic years, influenza activity alternated between influenza A and B viruses. Early epidemics were predominantly associated with A(H3N2), followed by a shift toward A(H1N1)pdm09 in 2018 with concurrent circulation of B/Yamagata. In 2019, B/Victoria became the predominant lineage, while B/Yamagata was no longer detected.

During the pandemic period, viral diversity was reduced. In 2020, A(H3N2) accounted for the majority of detected viruses, whereas in 2021, only B/Victoria was identified. In 2022, B/Victoria remained predominant with limited re-emergence of A(H3N2); A(H1N1)pdm09 was not detected during this period (Supplementary Table S1).

In the post-pandemic period, influenza A viruses again predominated. A(H3N2) was the dominant strain in 2023, with concurrent circulation of A(H1N1)pdm09. In 2024, B/Victoria and A(H1N1)pdm09 co-dominated, accompanied by a reduction in the proportion of A(H3N2). Across the entire study period, the B/Yamagata lineage was not detected after 2020 (Figure 2).

#### 3.2.2 Changes in seasonality and epidemic characteristics

**Figure 3:**
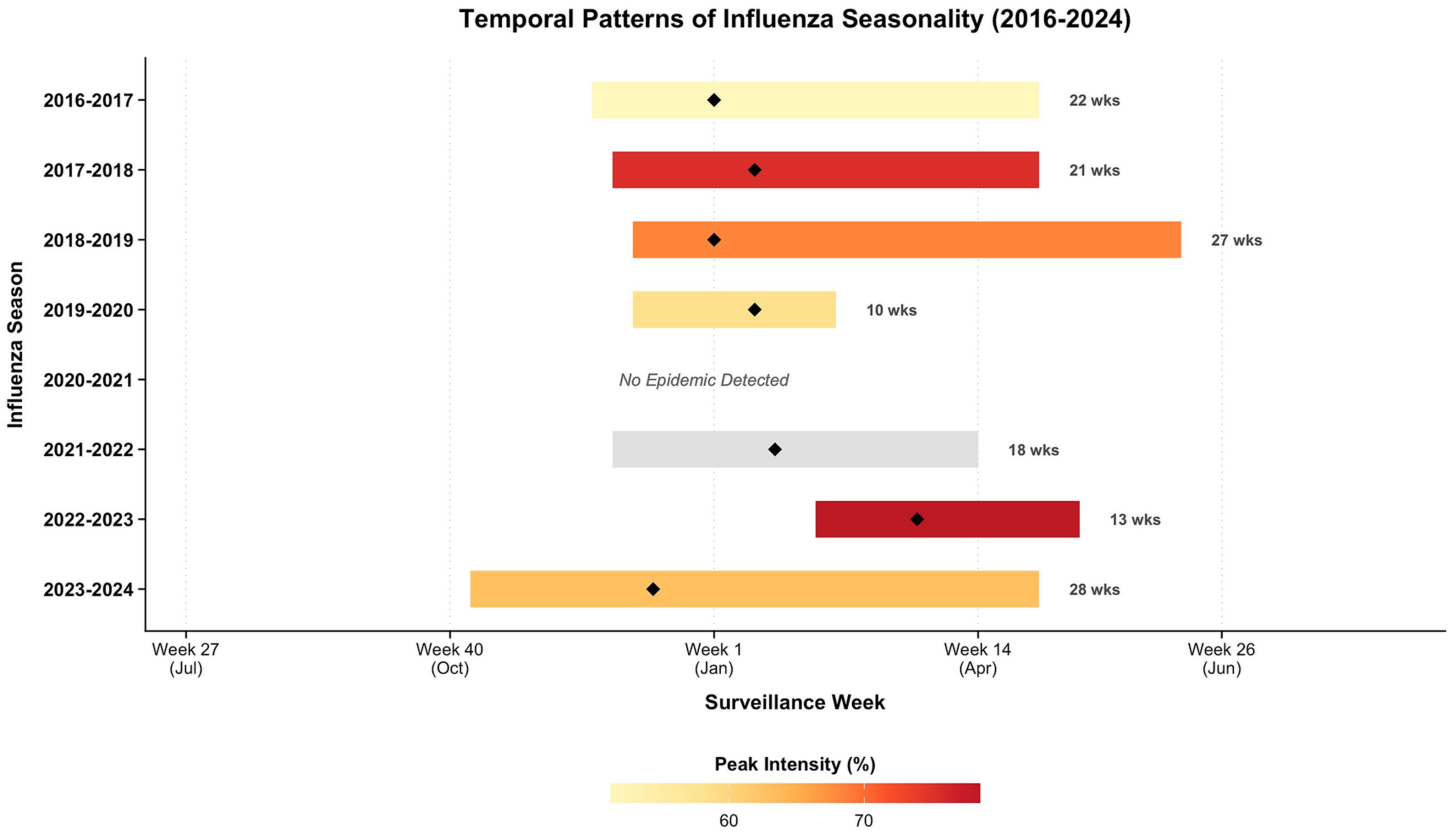
Characterization of influenza seasonality duration, timing, and peak intensity from 2016 to 2024. This figure visually presents the key epidemiological characteristics of influenza seasons, including their duration, timing, and peak intensity, over an eight-year period. Horizontal bars delineate the duration of each epidemic season, defined by onset and termination weeks. The length of each bar corresponds to the seasonal duration in weeks (values annotated on the right). Black diamonds (◆) indicate the specific week of peak intensity. The color gradient of the bars maps to the magnitude of peak intensity, ranging from lower intensity (light yellow) to higher intensity (dark red).

Figure 3 illustrates the temporal evolution of epidemic intervals, peak timing, and intensity throughout the study period. During the pre-pandemic phase, seasonal patterns exhibited stability with epidemic windows typically spanning 21 to 27 weeks. Activity consistently peaked in early January, maintaining moderate to high intensity levels (Figure 3).

This regularity was disrupted by the pandemic, evidenced by the abrupt truncation of the 2019–2020 season to 10 weeks and the complete absence of a detectable epidemic curve in 2020–2021. The subsequent resumption of activity in the 2021–2022 season was characterized by a contracted 18-week duration and substantially reduced intensity (Figure 3).

The post-pandemic recovery period was characterized by pronounced alterations in influenza seasonal timing and intensity. The 2022–2023 season presented as a delayed, high-intensity spring wave, with its peak shifting significantly toward mid-March, a notable departure from the typical January baseline. Despite being the shortest observed season at 13 weeks, it registered the highest peak intensity throughout the study period. In contrast, the 2023–2024 season marked a return to an early winter onset, yet with a substantially expanded duration. This season spanned an unprecedented 28 weeks, with peak activity occurring in December, approximately 1 month earlier than the pre-pandemic January baseline (Figure 3).

To assess the robustness of our epidemic season definitions, a sensitivity analysis was performed using the alternative proportion of thresholds of 5 and 15%. This analysis confirmed that our main findings regarding altered seasonality and epidemic timing were highly robust, with epidemic peak timings remaining identical across all thresholds (Supplementary Table S2; Supplementary Figures S4, S5).

### 3.3 Age-specific distribution of influenza burden across epidemic periods

#### 3.3.1 Changes in total case counts and age composition

**Figure 4:**
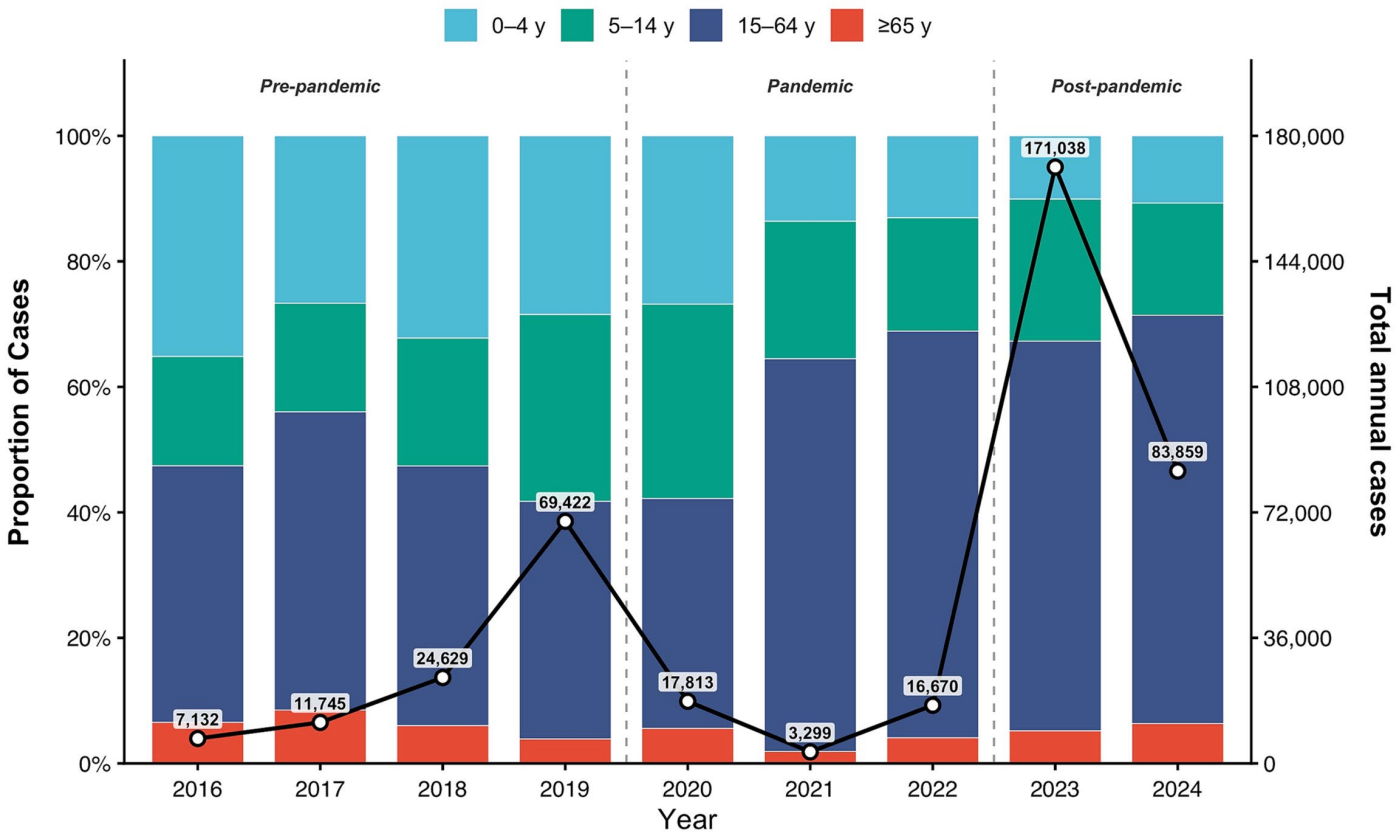
Age-stratified Proportional Distribution of Influenza Cases and Annual Total Case Counts across Epidemiological Phases, 2016–2024. This figure illustrates the annual total number of influenza cases and their proportional distribution across different age groups (0–4 years, 5–14 years, 15–64 years, ≥65 years) from 2016 to 2024. This proportional breakdown is represented by stacked bar charts, referenced against the left y-axis. Dashed vertical lines delineate the study period into three distinct epidemiological phases: pre-pandemic (2016–2019), pandemic (2020–2022), and post-pandemic (2023–2024). Numerical annotations above the line graph indicate the total influenza cases for each respective year.

Figure 4 illustrates the temporal dynamics of aggregate case counts and age-specific proportions. Total case numbers followed a distinct “increase-suppression-resurgence” trajectory. After peaking at 69,422 cases in 2019, activity was sharply suppressed during the pandemic, reaching a nadir of 3,299 cases in 2021. The post-pandemic phase witnessed a dramatic rebound, with cases surging to a historical high of 171,038 in 2023 before moderating to 83,859 in 2024 (Figure 4).

Regarding age composition, children aged 0–14 years constituted the majority of cases prior to the pandemic. The pandemic phase saw a marked demographic shift as adults (15–64 years) expanded their proportional share, becoming the predominant group in 2021 and 2022. In the post-pandemic phase, adults (15–64 years) retained the highest proportion of cases, although school-aged children (5–14 years) also experienced a notable resurgence in their contribution to the total burden (Figure 4).

#### 3.3.2 Temporal trends in incidence and relative risk by age group

**Figure 5:**
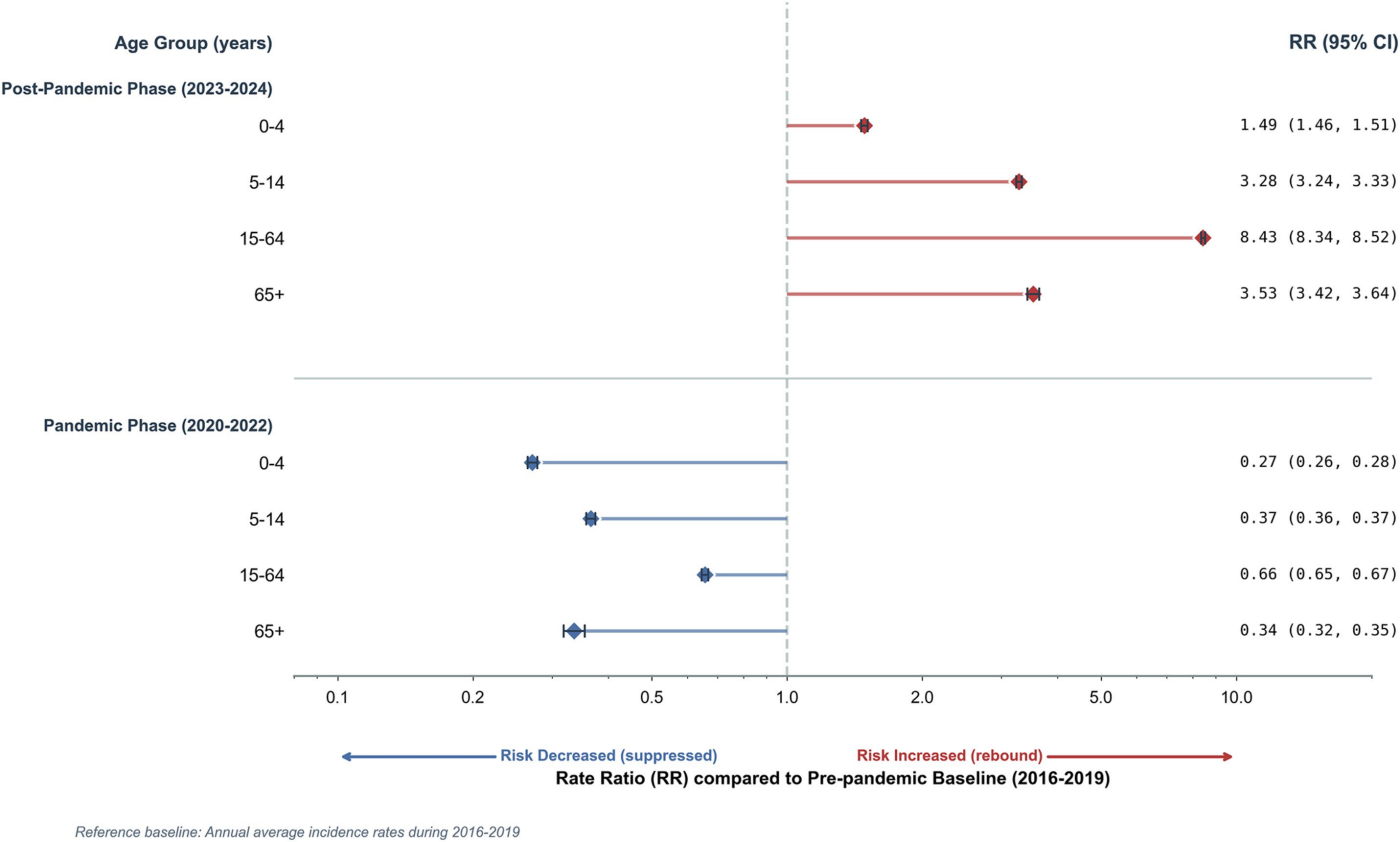
Age-stratified rate ratios (RR) of influenza incidence during the pandemic and post-pandemic periods. This figure summarizes the age-specific changes in influenza incidence risk during the pandemic and post-pandemic phases, compared to pre-pandemic baseline levels. Changes in influenza risk are shown as rate ratios (RR) relative to the 2016–2019 baseline. The left panel depicts the suppression effect on influenza incidence during the pandemic phase, while the right panel illustrates the rebound effect and elevated risk during the post-pandemic phase (2023–2024). Error bars represent 95% confidence intervals (CI). An RR < 1 indicates a reduced risk (suppression) compared to baseline, whereas an RR > 1 indicates an elevated risk (rebound). Note: The *x*-axis is presented on a logarithmic scale.

Age-specific analysis reveals distinct patterns in both absolute incidence and relative risk relative to the 2016–2019 baseline (Figure 5; Supplementary Table S3). Pre-pandemic trends showed increasing incidence peaking in 2019, with children (0–14 years) consistently exhibiting higher rates than other groups. The 0–4 age group recorded the highest rate (15,505 per 100,000), followed by the 5–14 age group (10,258 per 100,000; Supplementary Table S3).

During the pandemic phase, incidence dropped precipitously across all age groups, with relative risks falling significantly below baseline levels (RR < 1). The most profound reduction occurred in young children (0–4 years; RR = 0.27, 95% CI: 0.26–0.28), followed by people over 65 (RR = 0.34) and school-aged children (RR = 0.37; Figure 5).

Post-pandemic trends were characterized by a divergence between absolute burden and relative growth. In 2023, absolute incidence surged to a historical high of 16,099 per 100,000 among school-aged children (5–14 years), surpassing their 2019 peak (Supplementary Table S3). While this group maintained the highest absolute incidence, adults (15–64 years) exhibited the most pronounced post-pandemic elevation in relative risk (RR = 8.43; 95% CI: 8.34–8.52), significantly exceeding that of people over 65 (RR = 3.53) and school-aged children (RR = 3.28). In contrast, the 0–4 age group showed the most modest relative increase (RR = 1.49; Figure 5).

## 4 Discussion

### 4.1 Overview of main findings

Longitudinal sentinel surveillance spanning 9 years characterizes the post-pandemic epidemiological landscape of influenza in a representative megacity. The data reveal a distinct “suppression–rebound” trajectory, with resurgence intensity notably exceeding historical baselines. Beyond record-high intensity and the persistent non-detection of the B/Yamagata lineage, we observed a divergence between absolute disease burden and relative risk growth. While school-aged children (5–14 years) continued to exhibit the highest absolute incidence, the post-pandemic resurgence was disproportionately driven by adults (15–64 years), who experienced a substantially greater relative increase in risk compared to pediatric populations.

### 4.2 Magnitude of resurgence and immunity gaps

The magnitude of this resurgence, characterized by historical highs in the proportion of ILI, is consistent with compensatory epidemics reported globally following the relaxation of non-pharmaceutical interventions (NPIs) (5, 13, 28), such as the intense 2022 season in Australia (29). This post-suppression “overshoot” is conceptually consistent with the notion of “immunity debt,” whereby prolonged interruption of viral circulation may result in an expanded pool of susceptible individuals (30, 31). The empirical results presented here align with modeling projections that anticipated multi-fold increases in peak intensity after extended periods of transmission suppression (32, 33). Furthermore, the rapid convergence of observed incidence toward counterfactual estimates in the interrupted time series (ITS) analysis suggests that influenza transmission dynamics exhibit intrinsic resilience, enabling a rapid rebound once population-level susceptibility is restored (33, 34). However, a critical distinction separates epidemiological patterns from their biological mechanisms. While the pronounced rebound and its congruence with counterfactual scenarios strongly support an “immunity gap” that mirrors theoretical immunity debt models, this metric serves as an epidemiological proxy, not a direct biological measurement. It is derived from the ILI surveillance data, a syndromic indicator that can be influenced by multiple factors beyond true viral circulation, including shifts in healthcare-seeking behaviors and clinical awareness, especially in the post-pandemic context (35, 36). Without concurrent serological data, the “immunity gap” in this study remains an inferred construct rooted in transmission dynamics; thus, the exact magnitude of immune erosion at the individual or population level cannot be biologically quantified. Seroprevalence data would be indispensable for directly assessing antibody titers and cellular immunity, thereby offering a definitive measure of susceptibility. Consequently, while our analysis offers robust epidemiological evidence of a rebound consistent with immunity debt theories, it underscores the urgent need for integrated seroepidemiological surveillance to fully characterize the biological underpinnings of such post-pandemic resurgences.

### 4.3 Altered viral landscape and epidemic kinetics

The post-pandemic viral landscape has undergone a notable reconfiguration, most prominently reflected in the sustained non-detection of B/Yamagata lineage circulation. Consistent with global surveillance reports (9, 37), no B/Yamagata viruses were detected after early 2020 in our sentinel system. This sustained non-detection is potentially attributable to the lineage’s lower effective reproductive number combined with the severe transmission bottleneck imposed by prolonged NPIs (37, 38). Although our data are derived from district-level surveillance, the sustained non-detection of B/Yamagata lineage is consistent with broader global observations and aligns with the rationale behind the World Health Organization’s recommendation to transition to trivalent influenza vaccine formulations (39). Concurrently, epidemic kinetics exhibited substantial variability, with seasonal durations ranging from compressed 13-week outbreaks to prolonged epidemics extending up to 28 weeks. This heterogeneity suggests that the sequential circulation of multiple influenza types/subtypes and lineages—such as A(H3N2) followed by B/Victoria—interacting with uneven population immunity profiles, can markedly extend the effective transmission window. Similar biphasic patterns driven by strain turnover have been reported in other temperate regions (40, 41), underscoring the role of viral interference in shaping epidemic kinetics (9). Such temporal instability underscores the importance of adaptive, real-time surveillance systems capable of capturing shifts in epidemic duration and viral composition, thereby informing a more accurate understanding of evolving influenza risk dynamics.

### 4.4 The burden of demographic shift on working-age adults

Post-pandemic influenza epidemiology has shifted markedly in this urban setting. While absolute incidence remained highest among children (<15 years), working-age adults (15–64 years) experienced a disproportionate increase in reported risk: 8.43-fold compared to 3.28-fold in children during the 2023–2024 season. This substantial disparity in relative risk expansion represents a departure from traditional influenza patterns, where children have historically been recognized as primary drivers of incidence.

Understanding this demographic shift requires consideration of several overlapping mechanisms, which can be broadly categorized into three domains: surveillance and behavioral factors, host-level biological and clinical modifiers, and population immunity dynamics.

First, surveillance dynamics and shifts in healthcare-seeking behavior likely constitute the primary drivers. The study period (2016–2024) spanned the pre-pandemic, pandemic, and post-pandemic eras. While the national ILI case definition and influenza diagnostic criteria (WS 285-2008) remained formally unchanged during this period (17, 22), the COVID-19 pandemic substantially altered healthcare-seeking behaviors and laboratory testing priorities between 2020 and 2022. Notably, heightened public health awareness following COVID-19 may have prompted adults to seek care for mild respiratory symptoms at higher rates. This interpretation is supported by age-specific differences in healthcare-seeking patterns: whereas pediatric care-seeking is predominantly parent-mediated and historically stable (42), behavioral shifts among working-age adults are likely far more pronounced. Concurrent improvements in diagnostic capacity and clinician vigilance could further amplify reported case counts.

This surveillance complexity is further amplified by the syndromic nature of ILI reporting and the presence of co-circulating pathogens. Co-infections (e.g., concurrent influenza with SARS-CoV-2 or RSV) are known to exacerbate clinical severity, with evidence showing significantly higher risks of severe outcomes and mortality compared to single infections (43, 44). While overall co-infection rates among routine outpatient respiratory illnesses remain relatively low (45, 46), the greater clinical severity associated with co-infections increases the likelihood that affected patients will be identified and tested within sentinel hospital networks, as clinical decision-making for multi-pathogen testing is known to be driven by disease severity (47). This phenomenon has the potential to introduce surveillance bias, subtly inflating the reported proportion of influenza during periods of intense pathogen co-circulation.

Second, host-level biological and clinical modifiers may further amplify the observed disparity. Specifically, prior SARS-CoV-2 infection and its associated post-acute sequelae (PASC, or Long COVID) may induce prolonged immune dysregulation and residual respiratory mucosal damage, potentially increasing individual susceptibility to subsequent respiratory pathogens (48–50). Moreover, these persistent symptoms (e.g., chronic cough, fatigue) overlap significantly with ILI criteria, complicating the interpretation of surveillance signals (49, 51). While standard virological surveillance effectively captures population-level influenza trends, individual clinical evaluation often encounters complex scenarios where symptoms overlap with other respiratory conditions. For these challenging cases, advanced imaging tools can be highly valuable for differential diagnosis. Modalities ranging from established CT-based approaches (52, 53) to emerging functional assessments, such as hyperpolarized propane MRI (54), provide detailed visualization of regional lung ventilation and structural abnormalities. Integrating these imaging techniques into clinical diagnostic workflows helps distinguish acute viral pneumonia from secondary complications or residual post-viral sequelae, thereby guiding more precise patient management.

Closely related to baseline health are individual-level determinants—such as underlying immune diseases, chronic conditions, elevated BMI, and smoking status. These well-documented modifiers of respiratory infections are far more prevalent in working-age adults than in children. Crucially for syndromic surveillance, these factors not only influence biological susceptibility but also significantly lower the threshold for seeking medical care. For instance, immunocompromised individuals or those with underlying chronic conditions (e.g., diabetes, cardiovascular diseases) are more prone to consult healthcare providers for mild-to-moderate respiratory symptoms, making them disproportionately captured by sentinel hospital networks (55, 56). Similarly, factors like elevated BMI and active smoking have been associated with prolonged or more pronounced respiratory symptoms, potentially driving higher outpatient consultation rates (57–59). Furthermore, the COVID-19 pandemic disrupted continuity of care for many patients with chronic conditions—including reduced medication adherence and delayed specialist consultations (60)—which may have cumulatively amplified the symptomatic burden among working-age adults during the post-pandemic resurgence, prompting increased healthcare utilization.

Finally, shifts in population immunity dynamics may constitute a critical underlying biological factor. Adults rely primarily on immune memory sustained through recurrent seasonal influenza exposures, whereas children develop immunity *de novo* (61, 62). Multi-year suppression of influenza circulation during the pandemic, combined with ongoing antigenic drift, could erode accumulated adult immunity—a process termed “immune waning” (63, 64). Compounding this vulnerability, influenza vaccination coverage among working-age adults (18–59 years) in Beijing remains exceedingly low (estimated at 0.8% during the 2021–2022 season) (65, 66), representing a structural feature of the city’s immunization program that, in the context of documented immune waning, warrants consideration as a potential contributing factor to the observed post-pandemic risk elevation in this age group—though this hypothesis requires prospective serological validation.

### 4.5 Limitations

Several limitations of this study warrant consideration.

First, data quality and measurement constraints introduce potential bias at the source. The ILI indicator captures a broad spectrum of co-circulating respiratory pathogens rather than influenza-specific transmission. In accordance with CISDCP reporting standards, clinically diagnosed and laboratory-confirmed cases are merged, and evolving testing practices alongside shifting public health priorities over the nine-year study period have inevitably introduced temporal heterogeneity and reporting bias. Furthermore, the aggregated nature of the dataset precludes access to individual-level clinical characteristics—including comorbidities, BMI, smoking status, and underlying immune conditions—that would be necessary to quantitatively disentangle comorbidity-driven healthcare utilization from genuine shifts in viral transmission dynamics.

Second, causal inference is substantially limited by the absence of concurrent biological and behavioral data. The observed post-pandemic surges across age groups are likely confounded by shifts in healthcare-seeking behavior and heightened clinical awareness, the precise magnitude of which cannot be measured without concurrent behavioral surveys. The immune waning hypothesis remains an inferred epidemiological construct, as the absence of serological data precludes biological quantification of population-level immune erosion. Accordingly, the pediatric relative risk patterns serve as an observational reference rather than a validated epidemiological baseline, and the narrative comparisons across age groups should be regarded as hypothetical explorations rather than verified conclusions.

Third, the generalizability of findings requires careful consideration. The overall post-pandemic ILI resurgence in Chaoyang District aligns closely with national surveillance data from the Chinese National Influenza Center (CNIC) (67, 68), suggesting that the broad shift in influenza activity reflects a nationwide trend. However, the disproportionate surge among working-age adults cannot currently be validated against national-level age-stratified data due to availability constraints, and whether this demographic pattern represents a localized phenomenon or a broader nationwide trend remains an open question warranting further investigation.

Future prospective studies should address these limitations by integrating individual-level behavioral surveys, serological assessments, and comprehensive clinical profiles to better elucidate the complex determinants underlying the observed epidemiological shifts.

## 5 Conclusion

This long-term analysis of population-level influenza syndromic surveillance in a major urban district suggests that the COVID-19 pandemic was associated with a complex epidemiological shift, rather than a simple return to historical patterns. Post-pandemic influenza activity in Chaoyang District has been characterized by altered syndromic intensity, a marked redistribution of reported cases toward working-age adults, and the sustained absence of B/Yamagata lineage detections—the latter aligning with global reports of its probable extinction. This demographic redistribution likely reflects a complex interplay of post-pandemic changes in healthcare-seeking behavior, surveillance sensitivity, and host-level biological factors; these interpretations should be regarded as hypothesis-generating rather than causally established. Population-level immune waning may also have contributed, though its relative impact cannot be quantified from syndromic surveillance data alone. Together, these findings underscore the value of age-stratified, real-time influenza monitoring and highlight the need for future studies integrating serological, virological, behavioral, and clinical data to better characterize the determinants of shifting influenza burden in the post-pandemic era.

## Data Availability

The data analyzed in this study is subject to the following licenses/restrictions: The raw data supporting the conclusions of this article will be made available by the authors, subject to the constraints of applicable laws and policies. Requests to access these datasets should be directed to the corresponding author.
