# Supplementary figures and images for: Suppression and resurgence: the evolving epidemiology of seasonal influenza from 2015 to 2024 in a core urban district of Beijing, China
Source: Front Public Health. 2026 May 14;14:1800701. doi: 10.3389/fpubh.2026.1800701 (PMC13216027; doi:10.3389/fpubh.2026.1800701)

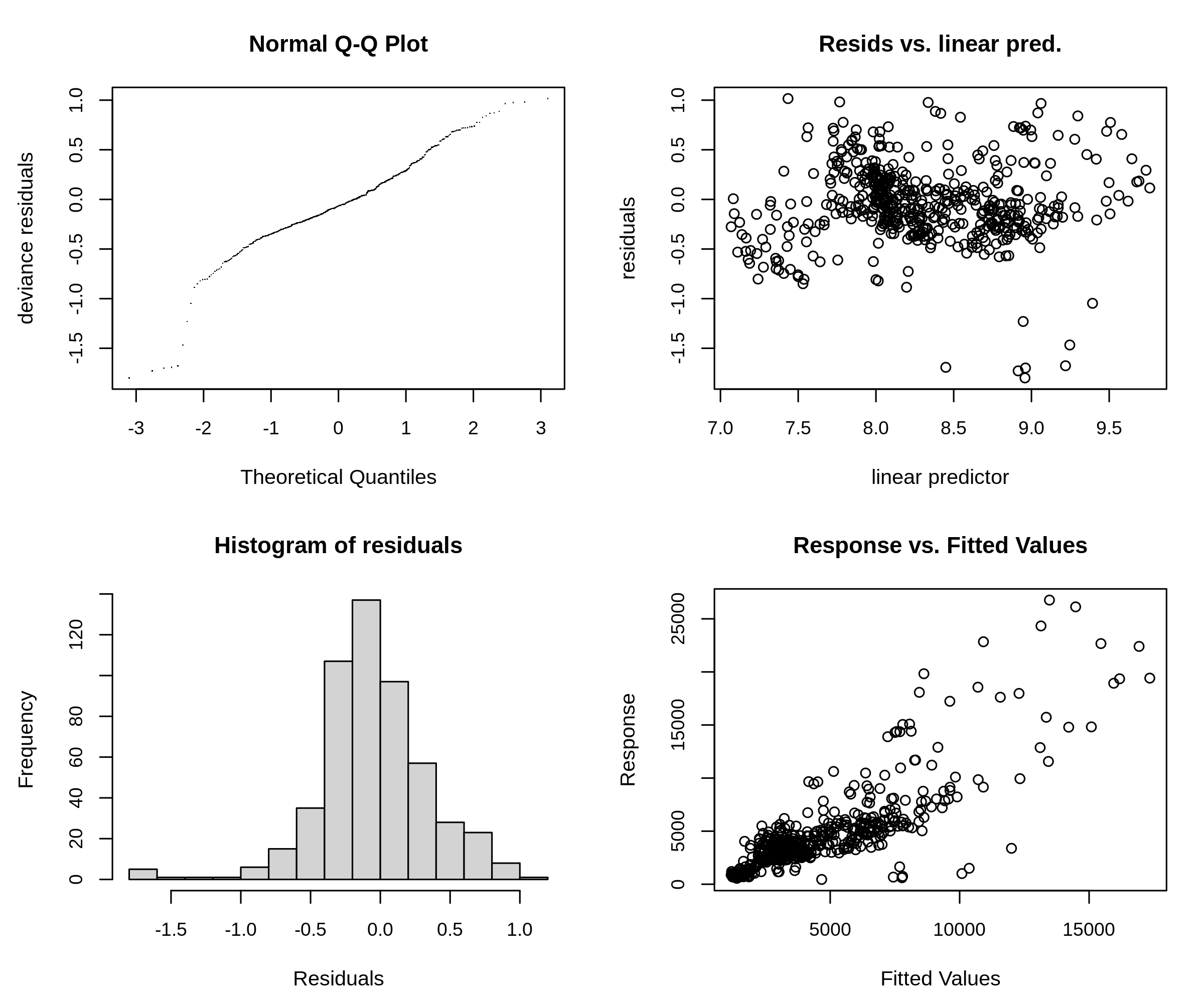

Supplement: Supplementary file 1 [file Image_1.JPEG]

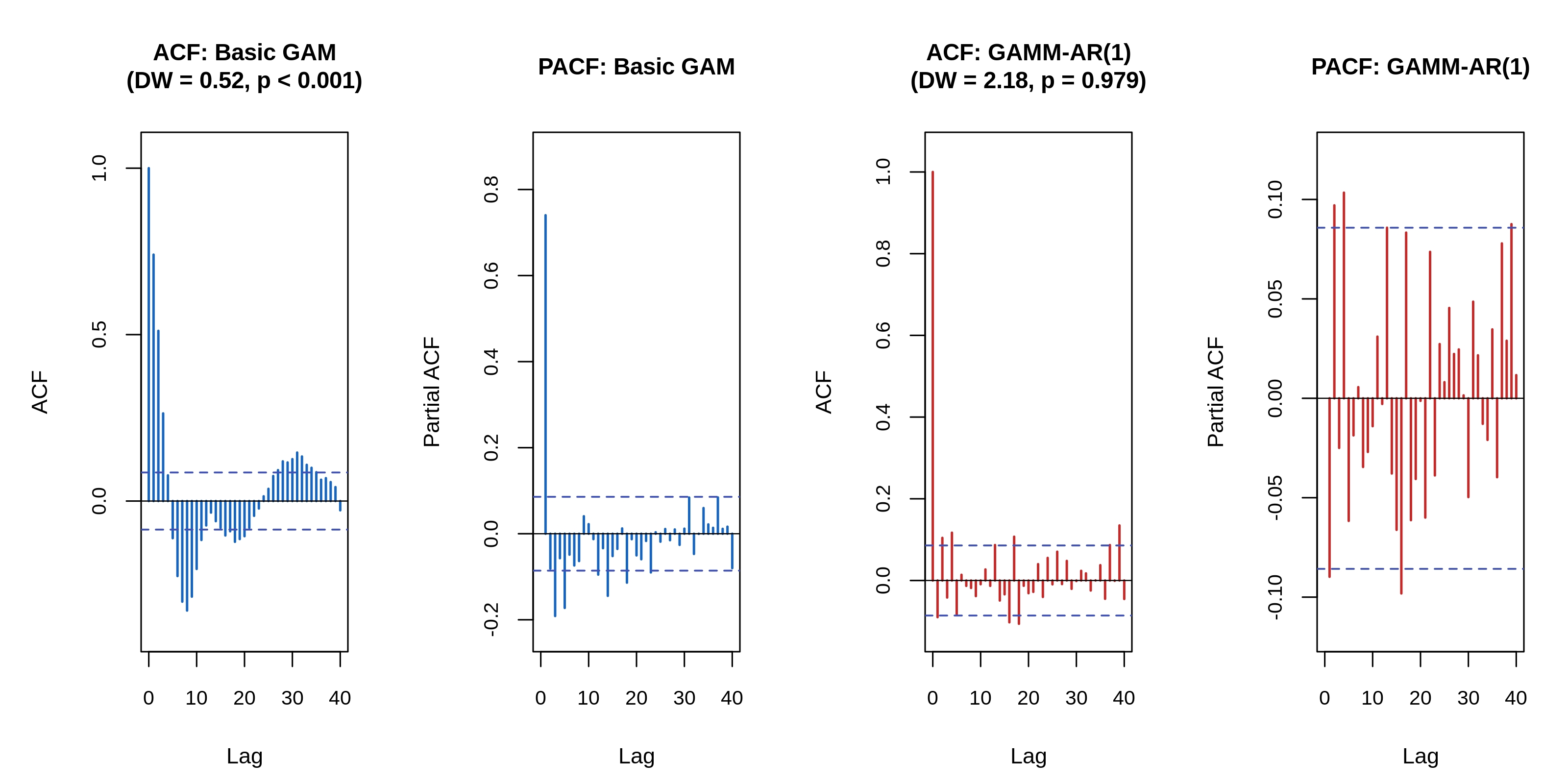

Supplement: Supplementary file 2 [file Image_2.JPEG]

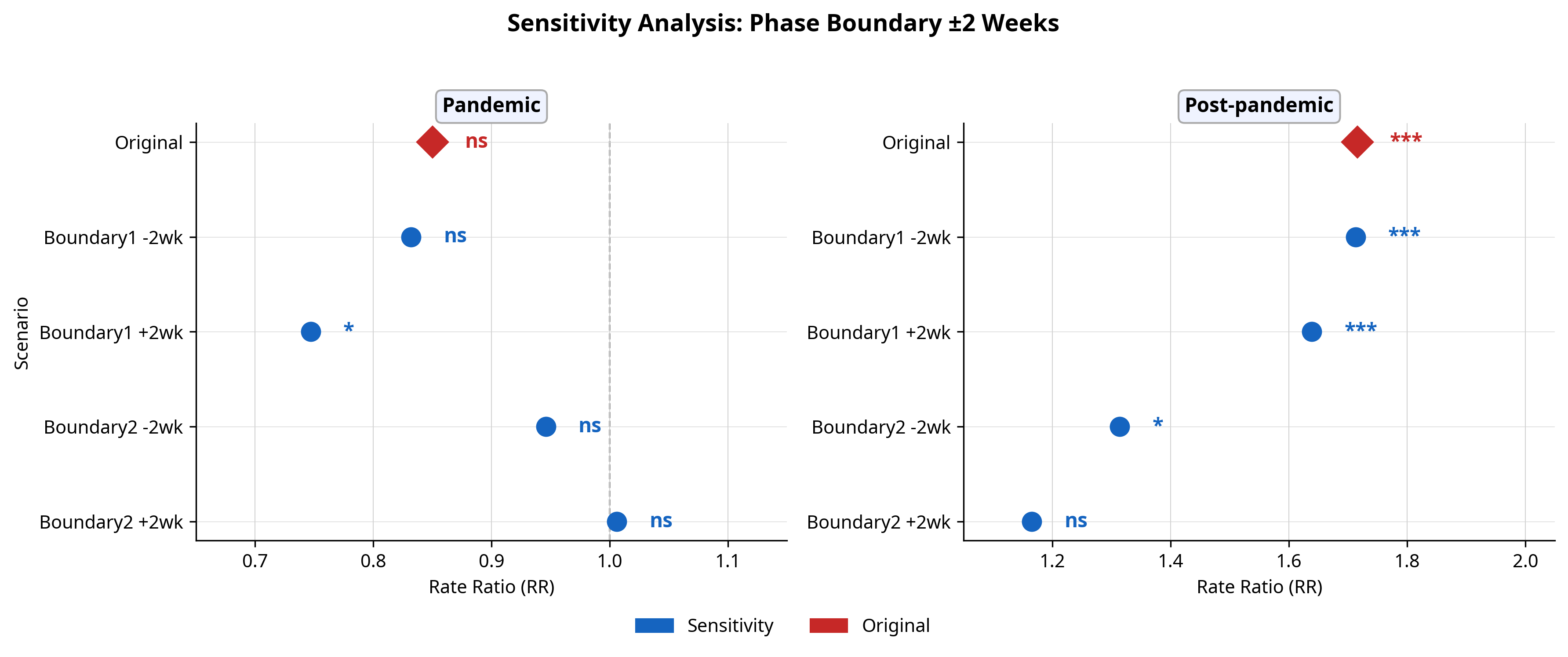

Supplement: Supplementary file 3 [file Image_3.JPEG]

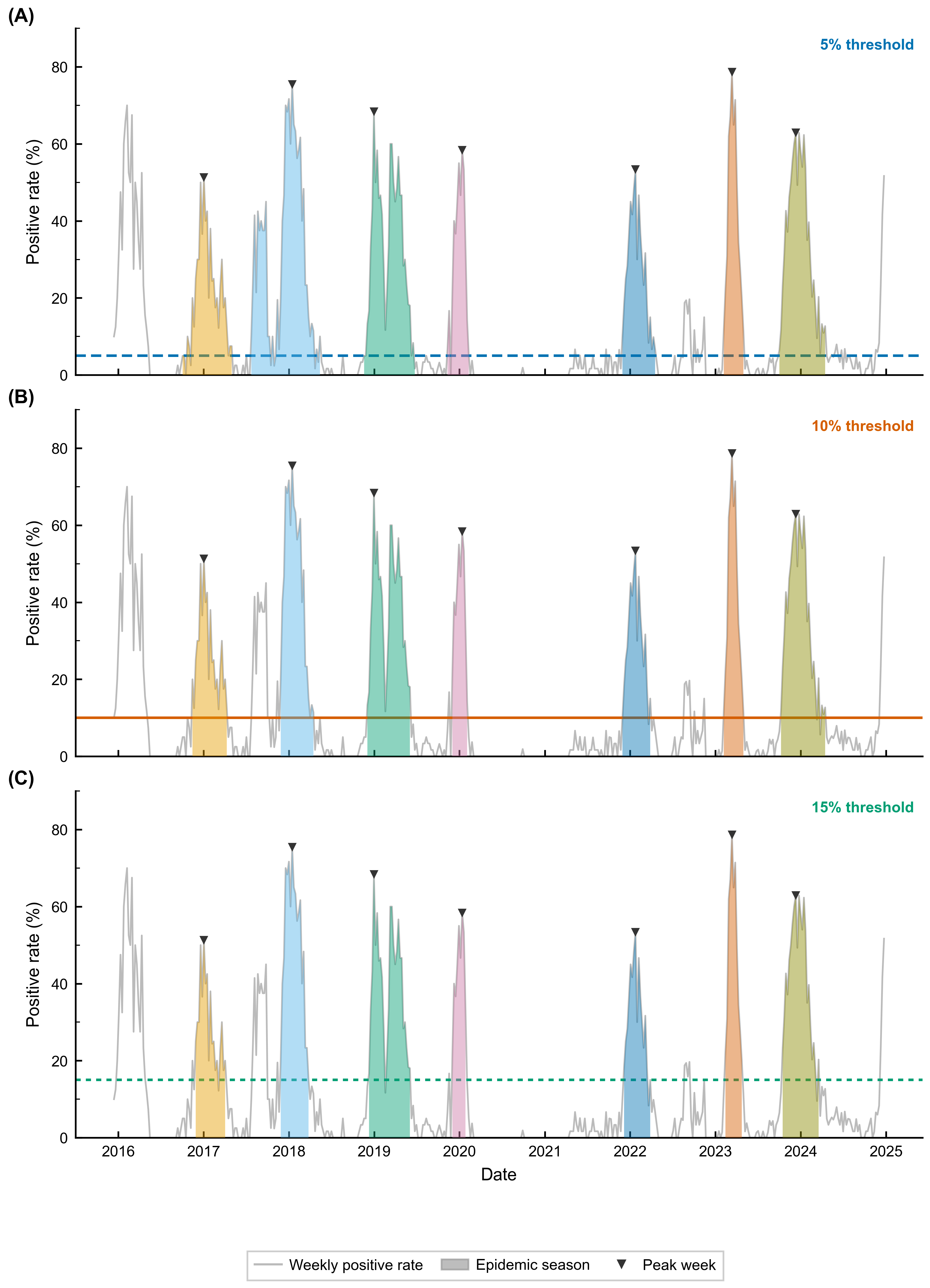

Supplement: Supplementary file 4 [file Image_4.PNG]

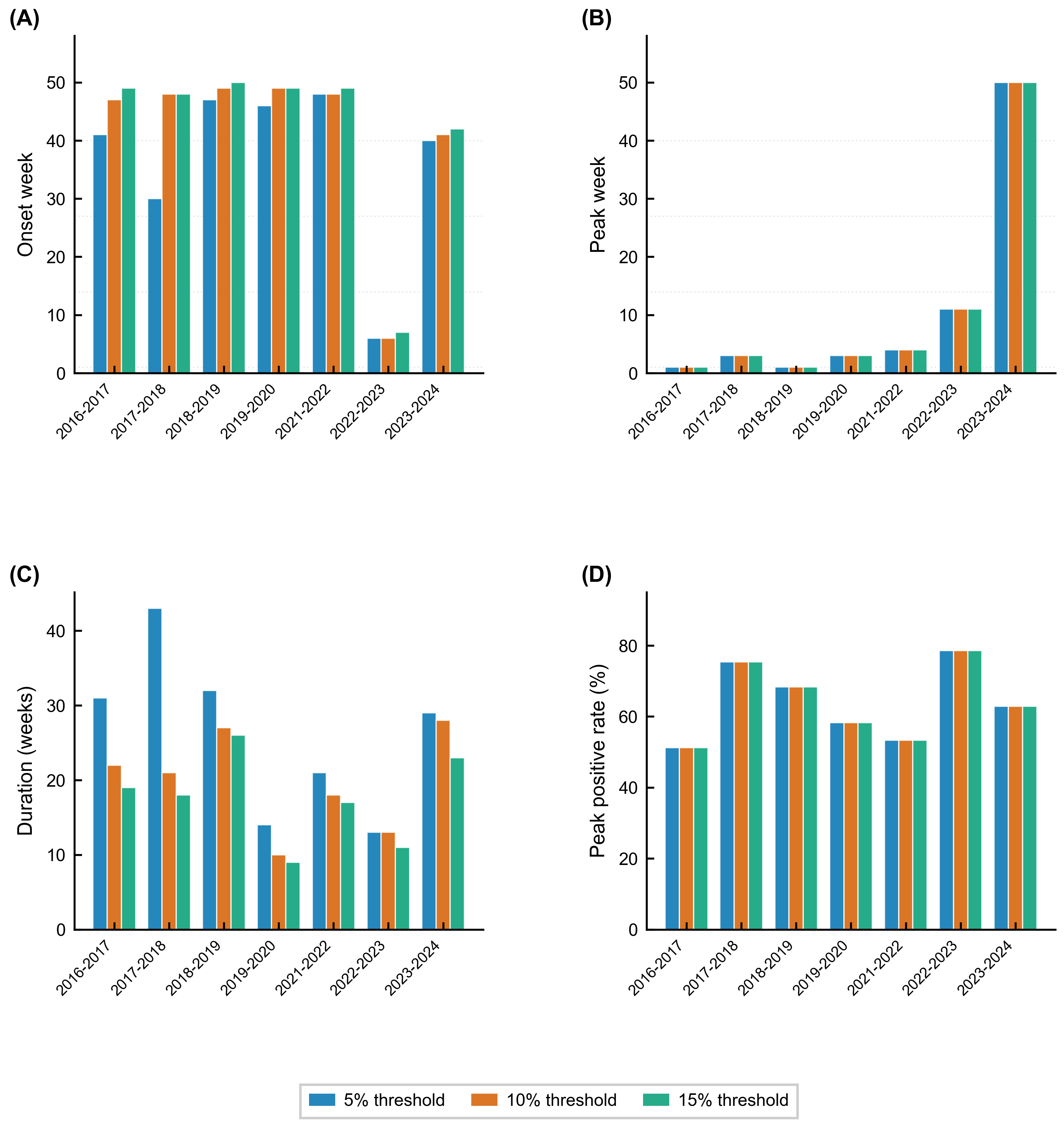

Supplement: Supplementary file 5 [file Image_5.PNG]
