# Supplementary material for: Suppression and resurgence: the evolving epidemiology of seasonal influenza from 2015 to 2024 in a core urban district of Beijing, China
Source: Front Public Health. 2026 May 14;14:1800701. doi: 10.3389/fpubh.2026.1800701 (PMC13216027; doi:10.3389/fpubh.2026.1800701)
Supplement: Supplementary file 6 [file Table_1.DOCX]

| Year | A(H1N1)pdm09(%) | A(H3N2)(%) | B/Victoria(%) | B/Yamagata(%) |
| --- | --- | --- | --- | --- |
| 2016 | 13.19 | 43.65 | 36.45 | 6.71 |
| 2017 | 20.53 | 59.34 | 2.26 | 19.1 |
| 2018 | 44.02 | 17.76 | 5.41 | 32.82 |
| 2019 | 24.28 | 30.92 | 44.8 | 0 |
| 2020 | 9.26 | 70.37 | 20.37 | 0 |
| 2021 | 0 | 0 | 100 | 0 |
| 2022 | 0 | 24.17 | 75.83 | 0 |
| 2023 | 24.38 | 69.59 | 6.03 | 0 |
| 2024 | 35.51 | 10.11 | 54.16 | 0 |

Supplementary table 1. Distribution of influenza subtypes and lineages, 2016-2024.
