# Supplementary material for: Suppression and resurgence: the evolving epidemiology of seasonal influenza from 2015 to 2024 in a core urban district of Beijing, China
Source: Front Public Health. 2026 May 14;14:1800701. doi: 10.3389/fpubh.2026.1800701 (PMC13216027; doi:10.3389/fpubh.2026.1800701)
Supplement: Supplementary file 7 [file Table_2.DOCX]

**Supplementary Table S2.** Comparison of influenza epidemic season characteristics across three positivity rate thresholds (5%, 10%, and 15%) using a consecutive two-week criterion, surveillance years 2016–2024.

| **Season** | **5% Threshold** | | | | | **10% Threshold** | | | | | | **15% Threshold** | | | | | |  |
| --- | --- | --- | --- | --- | --- | --- | --- | --- | --- | --- | --- | --- | --- | --- | --- | --- | --- | --- |
|  | **Onset Week** | **Peak Week** | **End Week** | **Duration (weeks)** | **Peak Rate (%)** | | **Onset Week** | **Peak Week** | **End Week** | **Duration (weeks)** | **Peak Rate (%)** | | **Onset Week** | **Peak Week** | **End Week** | **Duration (weeks)** | **Peak Rate (%)** | |
| **2016-2017** | 41 | 1 | 18 | 31 | **51.2** | | 47 | 1 | 15 | 22 | **51.2** | | 49 | 1 | 14 | 19 | **51.2** | |
| **2017-2018** | 30 | 3 | 20 | 43 | **75.4** | | 48 | 3 | 16 | 21 | **75.4** | | 48 | 3 | 13 | 18 | **75.4** | |
| **2018-2019** | 47 | 1 | 26 | 32 | **68.3** | | 49 | 1 | 23 | 27 | **68.3** | | 50 | 1 | 23 | 26 | **68.3** | |
| **2019-2020** | 46 | 3 | 7 | 14 | **58.3** | | 49 | 3 | 6 | 10 | **58.3** | | 49 | 3 | 5 | 9 | **58.3** | |
| **2020-2021** | — | — | — | 0 | **0.0** | | — | — | — | 0 | **0.0** | | — | — | — | 0 | **0.0** | |
| **2021-2022** | 48 | 4 | 16 | 21 | **53.3** | | 48 | 4 | 13 | 18 | **53.3** | | 49 | 4 | 13 | 17 | **53.3** | |
| **2022-2023** | 6 | 11 | 18 | 13 | **78.6** | | 6 | 11 | 18 | 13 | **78.6** | | 7 | 11 | 17 | 11 | **78.6** | |
| **2023-2024** | 40 | 50 | 16 | 29 | **62.9** | | 41 | 50 | 16 | 28 | **62.9** | | 42 | 50 | 12 | 23 | **62.9** | |

Note：Onset Week, Peak Week, and End Week are expressed as ISO week numbers within the surveillance year (Week 27 of year N to Week 26 of year N+1). Duration is expressed in weeks. Peak Rate (%) denotes the maximum weekly influenza positivity rate during the epidemic season. "—" indicates no epidemic season was identified for that threshold. The 2020–2021 season had no detectable epidemic, likely attributable to COVID-19 non-pharmaceutical interventions.
