# Supplementary material for: Suppression and resurgence: the evolving epidemiology of seasonal influenza from 2015 to 2024 in a core urban district of Beijing, China
Source: Front Public Health. 2026 May 14;14:1800701. doi: 10.3389/fpubh.2026.1800701 (PMC13216027; doi:10.3389/fpubh.2026.1800701)
Supplement: Supplementary file 8 [file Table_3.DOCX]

Supplementary table 3. Age-Specific incidence rate (per 100,000 population), 2015-2024.

| Year | 0-4 | 5-14 | 15-64 | ≥65 |
| --- | --- | --- | --- | --- |
| 2015 | 216 | 75 | 13 | 20 |
| 2016 | 1652 | 559 | 95 | 111 |
| 2017 | 2131 | 920 | 189 | 236 |
| 2018 | 5983 | 2455 | 359 | 346 |
| 2019 | 15505 | 10258 | 965 | 643 |
| 2020 | 3169 | 2260 | 255 | 201 |
| 2021 | 291 | 289 | 82 | 12 |
| 2022 | 1430 | 1220 | 431 | 127 |
| 2023 | 11611 | 16099 | 4288 | 1527 |
| 2024 | 6103 | 6276 | 2243 | 858 |
